# Toward Optimal Irrigation Management at the Plot Level: Evaluation of Commercial Water Potential Sensors

**DOI:** 10.3390/s23229255

**Published:** 2023-11-17

**Authors:** Alaitz Aldaz-Lusarreta, Miguel Ángel Campo-Bescós, Iñigo Virto, Rafael Giménez

**Affiliations:** 1 Institute for Innovation & Sustainable Development in Food Chain (IS-FOOD), Public University of Navarre (UPNA), Campus de Arrosadia, 31006 Pamplona, Spain; miguel.campo@unavarra.es (M.Á.C.-B.); inigo.virto@unavarra.es (I.V.); rafael.gimenez@unavarra.es (R.G.); 2 Department of Engineering, Los Olivos Building, Public University of Navarre (UPNA), Campus de Arrosadia, 31006 Pamplona, Spain; 3 Department of Science, Los Olivos Building, Public University of Navarre (UPNA), Campus de Arrosadia, 31006 Pamplona, Spain

**Keywords:** soil water dynamics, soil water measurement, soil water monitoring, agronomy, performance

## Abstract

Proper irrigation practice consists of applying the optimum amount of water to the soil at the right time. The porous characteristics of the soil determine the capacity of the soil to absorb, infiltrate, and store water. In irrigation, it is not sufficient to only determine the water content of the soil; it is also necessary to determine the availability of water for plants: water potential. In this paper, a comprehensive laboratory evaluation—accuracy and variability—of the world’s leading commercial water potential sensors is carried out. No such comprehensive and exhaustive comparative evaluation of these devices has been carried out to date. Ten pairs of representative commercial sensors from four different families were selected according to their principle of operation (tensiometers, capacitive sensors, heat dissipation sensors, and resistance blocks). The accuracy of the readings (0 kPa–200 kPa) was determined in two soils of contrasting textures. The variability in the recordings—repeatability and reproducibility—was carried out in a homogeneous and inert material (sand) in the same suction range. The response in terms of accuracy and value dispersion of the different sensor families was different according to the suction range considered. In the suction range of agronomic interest (0–100 kPa), the heat dissipation sensor and the capacitive sensors were the most accurate. In both families, registrations could be extended up to 150–200 kPa. The scatter in the readings across the different sensors was due to approximately 80% of the repeatability or intrinsic variability in the sensor unit and 20% of the reproducibility. Some sensors would significantly improve their performance with ad hoc calibrations.

## 1. Introduction

Proper irrigation practice in its different modalities—surface, sprinkler, and drip—basically consists of applying the optimum amount of water to the soil, at the right time, so that it can be used by plants. For this purpose, a water balance technique is generally used, which enables the prediction of the amount of water that the plant consumes (evapotranspiration) and the amount of available water, i.e., the water that can be readily used by the plant. The main limitation of this technique is that the evapotranspiration values are averages for the zone, which makes this type of irrigation strategy a rough estimation. On the other hand, there is another type of irrigation strategy more efficient and more appropriate for precision agriculture [1,2,3] based on the use of humidity sensors and water potential sensors [4,5,6], which allow continuous and in situ recording of, respectively, soil water content and its availability (suction or degree of retention in the soil matrix) for crops [7,8].

Soils are composed of mineral particles (sand, silt, and clay) and organic elements of different sizes and shapes. The proportion of each of these defines the soil texture. In turn, these particles are usually grouped in aggregates, also of varying size and shape, forming the soil structure. The texture and structure of each soil define its porosity. Soil is, in short, a complex, porous material. The characteristics of the soil pore system, especially the size (equivalent diameter) and shape of the dominant pores, ultimately determine the soil’s capacity to absorb, infiltrate, and store water [9].

Water content and suction are related through the well-known soil water retention curve (SWRC) [6,10,11]. Therefore, each soil type has a specific SWRC and is conditioned by its porous system and, therefore, by the texture, structure, and organic matter content present in the soil. In addition, the form of the SWRC may be slightly different depending on whether it is obtained by absorption or desorption (hysteresis phenomenon) [9].

The complexity of the phenomenon of absorption—and, therefore, of soil water availability—explains the great variety and types of water potential sensors present in the literature. As a result, there is no unequivocal—and even less universal—proposal for classifying these devices, at least to the best of our knowledge. Tentatively, and according to their principle of operation, we propose to group them into four large families: (i) tensiometers, (ii) capacitive sensors, (iii) heat dissipation sensors, and (iv) resistance blocks (see details below).

In this paper, a comprehensive laboratory evaluation—accuracy and variability—of ten of the world’s leading commercial water potential sensors is carried out. To the best of our knowledge, no such broad and comprehensive comparative evaluation of these devices has been carried out to date. It is worth mentioning the recent work by Jackisch et al. [12] in which the field performance of numerous water potential sensors is discussed; however, this assessment is basically limited to examining the consistency of measurements through cross-correlation analysis between the different devices. In fact, those authors clarify that the correlation value does not reflect per se the performance or reliability of the sensors studied.

## 2. Materials and Methods

### 2.1. Water Potential Sensors: Description and Setup

**Figure 1:**
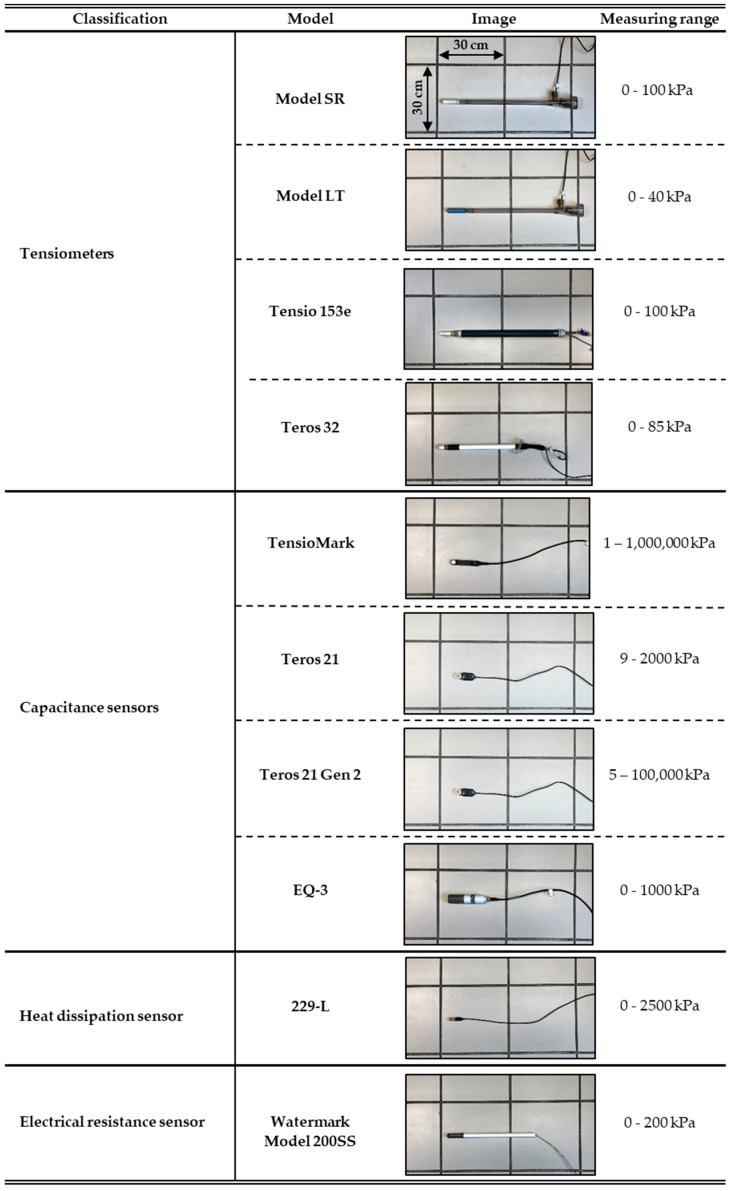
Classification, model, and measurement range of each commercial water potential sensor evaluated.

The sensors selected for the present investigation belong to four main families whose descriptions and operation principles are briefly explained below (Figure 1).

Tensiometers. A tensiometer device consists of a water reservoir, a porous ceramic capsule, and a vacuum gauge. When the tensiometer has been installed at the desired depth, the energy of the water inside the tensiometer is balanced with that of the surrounding soil. As the soil dries out, it draws more water from the reservoir through the porous capsule, creating a negative pressure (vacuum) inside the reservoir measured by the vacuum gauge [13,14,15].

Capacitive sensors. Capacitive sensors are composed of a porous ceramic with a known humidity vs. water potential ratio. A sensor that measures the water content of the ceramic is housed inside the ceramic. When the sensor is placed in the soil, the suction on the porous ceramic is balanced with the surrounding soil. In this way, the moisture content of the ceramic is measured and by means of the calibration curve (moisture vs. water potential ratio), the suction is inferred [9,13,14,16].

Heat dissipation sensors. Heat dissipation sensors consist of a heating element and a thermocouple placed inside a porous ceramic. An electric current pulse is sent and the thermocouple measures the temperature rise. The magnitude of the temperature rise varies with the amount of water in the porous ceramic, which changes as the soil becomes wetter and drier. The water potential of the soil is determined by applying a regression equation [13,14,16,17].

Resistance blocks. Resistance blocks consist of a pair of electrodes embedded in a porous block, which is buried. The electrical resistance between the electrodes of the porous block is proportional to its water content, which is, in turn, related to the water potential of the surrounding soil. As the soil, and thus the porous block, dries out, the electrical resistance decreases [14,18,19].

Then, 10 pairs of sensor models were selected so that each of the 4 sensor families was represented by at least one of these pairs (Figure 1).

Setup. The Teros 21, Teros 21 Gen 2, and Tensiomark capacitive sensors as well as the Teros 32 tensiometer were connected according to the SDI-12 protocol following the corresponding user manual [20,21,22,23]. This is a communications protocol that allows a sensor via a microprocessor to transfer measurement data to a datalogger. Normally, the sensor records a reading, the microprocessor performs calculations based on the raw sensor reading and transforms the reading into user-intelligible units—in this case, kPa and for the Tensiomark, pF—and finally, via the SDI-12 protocol, transfers the measurements to the datalogger [24]. The rest of the sensors were connected in analog form, so for the transformation of the raw reading, the transformation equations provided in each sensor manual were used [25,26,27,28,29]. In all sensors, the raw unit used was millivolt (mV), except for the heat dissipation sensor in which case the unit to transform was temperature difference (∆T, °C) [30].

It is important to mention that, in the case of the heat dissipation sensor (model 229-L), by applying the formula indicated in the manual [30] erroneous values were obtained; therefore, following the proposal of Reder et al. [31], a mathematical adjustment was made. This is why the results obtained with the 229-L sensor would be affected by this calibration and, therefore, this adjustment will have an impact on the comparison with the rest of the sensors under study.

Finally, it should be noted that, at the beginning of this research, the feasibility of all sensors was verified by testing their performance in air and water.

Tests were carried out with the selected sensors in order to evaluate (i) the accuracy of the readings and (ii) the variability (repeatability and reproducibility) in the readings.

### 2.2. Test 1: Sensor Accuracy Evaluation

#### 2.2.1. Soils

The sensors were evaluated using two contrasting soils (Table 1). Both soils were sieved at 2 mm.

Treatments and replicates. From the combination of 10 sensors and 2 soils, 20 treatments were defined, plus the control treatment (see below). The experiments—a combination of sensors and soils—were repeated twice, each repetition with a different sensor unit. In the case of the Teros 21 Gen 2 sensor (Figure 1), only one unit was available, so the same sensor unit was used in the repetitions.

#### 2.2.2. Experimentation

**Figure 2:**
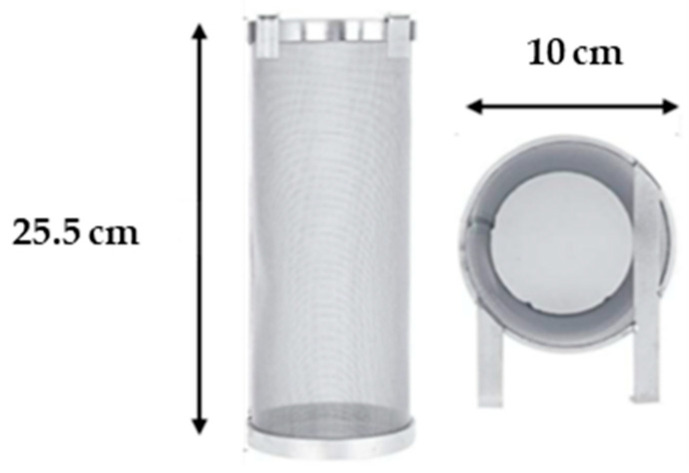
Stainless steel meshed cylinder used for sensor evaluation.

The evaluation of each of the commercial sensors was carried out in the laboratory in a volume of soil (bulk density = 1.16 g·cm^−3^) contained in a stainless steel mesh cylinder (Figure 2), in the center of which the sensor was housed. The following technical and operational criteria were taken into account in the selection of this container.

The container is meshed so that the evaporative demand is homogeneous over its entire surface, thus ensuring a uniform variation in soil moisture content during the experiment (see below). Greater homogeneity in water content is also possible owing to the cylindrical shape of the container since the distance from its center (where the sensor is located) to any point on its periphery is constant. Furthermore, the choice of the cylinder dimensions (Figure 2) took into account the dimensions of the sensors and their radius of action (approximately 2 to 3 cm).

Determination of reference values (control treatment). In order to compare the readings recorded by the sensors during the experiment (see below), it was necessary to establish reference suction values at different moisture contents in both soils, i.e., to determine, for each soil, its corresponding SWRC (in desorption). Then, during the experiment, in order to estimate the suction value in the soil at a given moment, it would be sufficient to measure the moisture content of the soil in order to infer the suction value from it.

The SWRCs of each soil were determined by using Richard’s Pressure Plates, as this is a more or less direct—and relatively simple and inexpensive considering the resources available—method of measuring suction at different degrees of humidity. By using these pressure plates, it is possible to apply positive pressure directly on the soil samples, unlike in commercial sensors where the suction reading would be somehow affected or conditioned by the presence of a certain synthetic porous material—specific to the sensor—in intimate contact with the soil. A clear example of the latter is the porous porcelain capsule of the tensiometers that conditions—due to its permeability—the movement of water from the water reservoir to the soil. It should be noted that the porous plate in Richard’s pot is basically a mere support for the soil samples; it also ensures that continuous water drainage from the soil sample is maintained during the measurement process.

The operating principle of this technique is, therefore, based on the injection of compressed air at a pressure equivalent to the water potential to be determined. When the pressure is applied, the ceramic plate allows the water extracted from the samples to drain through a collector until the equilibrium state is reached (the samples stop draining). The process ends with the weighing of the samples and their subsequent drying. In this way, and by weight difference, the water content of the sample is determined [32].

The soil for the determination of the SWRC was sieved at 2 mm and compacted to a bulk density of 1.16 g·cm^−3^; this treatment was also performed on the fill soil of the experimental cylinders (see above). In addition, the SWRC of each soil (Soil 1 and Soil 2, Table 1) was defined from 0 to 200 kPa as this is the range of interest for agronomic applications (e.g., for irrigation purposes). Water available to plants is mainly present at suctions below 100–150 kPa and, in addition, most conventional sensors (especially tensiometers) stop working (due to cavitation) at 100 kPa.

**Figure 3:**
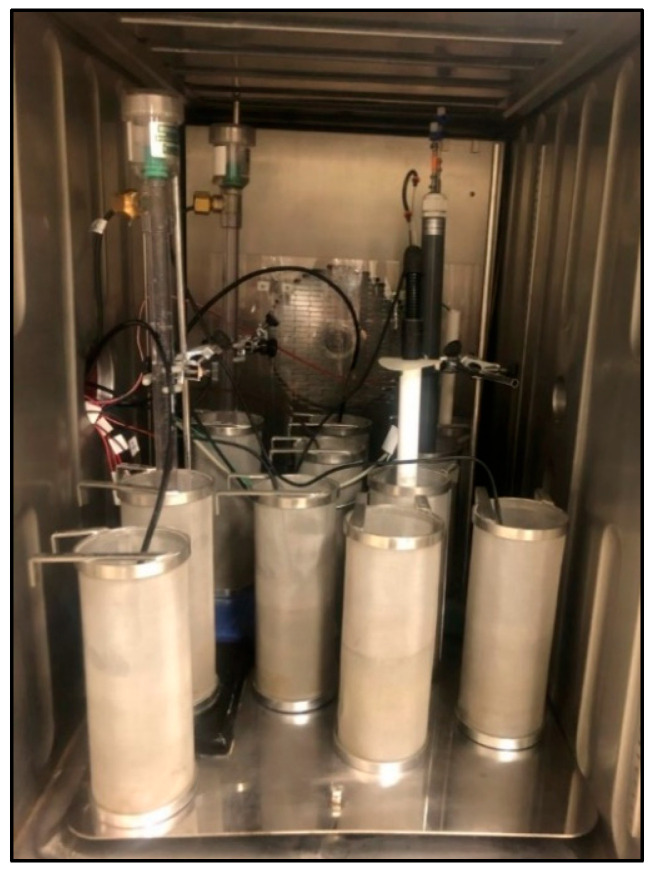
Cylinders used in the experiment (see Figure 2)—each one housing a specific sensor—placed inside the climatic chamber. Note: image prior to the placement of the control cylinder (explanation in the text).

Experimental design. Each experiment was carried out inside a large climatic chamber housing the different treatments (Figure 3), i.e., 10 meshed cylinders, each containing soil and a given sensor, plus an extra control cylinder (see below for more details on the latter).

In each case, the soil was brought to saturation (via capillary action). It was then forced to dry (desorption) under controlled conditions. Drying was carried out in a chamber where both humidity and ambient temperature could be controlled in order to establish a specific drying rate. Since each sensor has a certain response time (not indicated by the manufacturer) the drying time was adjusted to that which would be more or less expected in the upper layer of an agricultural soil, in situ. For example, a drying rate that could be expected in the first 10 cm of the soil profile in field conditions with a maize crop, in a month of high evapotranspiration, is 1.5 L·m^−3^·h^−1^ [33]. Preliminary tests determined that this rate of variation in moisture content was approximately reached at a relative humidity of 80% and a temperature of 10 °C.

Inside the climate chamber, the sensor for each treatment was connected to a datalogger, which allowed automatic data recording throughout the experiment.

The chamber also housed a cylinder containing only soil (control treatment, mentioned above) subjected to the same drying conditions as the other treatments. By weighing differences (precision of 0.01 g) of this cylinder, it was possible to determine the moisture content of the soil at a given moment. And with these data, the corresponding suction value (reference value, see above) could be inferred from the SWRC of the soil in question.

Experimental protocol. The experiment started with saturated soil (0 kPa). Approximately every hour, the suction value measured by each sensor and the weight of the control treatment were recorded to determine the moisture content. This was carried out until approximately 200 kPa was reached.

Data analysis. The sensors were evaluated using the dimensionless Nash Sutcliffe Efficiency Index (*NSE*) (Equation (1)), the root mean square error (*RMSE*) (Equation (2)), and the percent bias (*PBIAS*) (Equation (3)) analysis methods:

(1) $$NSE=1-\frac{\sum_{i=1}^{n}\;{\left(P_{i}-O_{i}\right)}^{2}}{\sum_{i=1}^{n}\;{\left(O_{i}-\bar{O}\right)}^{2}}$$

(2) $$RMSE=\sqrt{\frac{\sum_{i=1}^{n}\;{\left(P_{i}-O_{i}\right)}^{2}}{n}}$$

(3) $$PBIAS=\frac{\sum_{i=1}^{n}{(O}_{i}-P_{i\;})\times 100}{\sum_{i=1}^{n}\;(O_{i})}$$

where *O_i_* and *P_i_* represent the sample (sample size n) containing the observations and the model/reference value estimates, respectively, and *Ō* is the mean of the observed values.

The NSE efficiency is a normalized statistic that determines the relative magnitude of the residual variance (reference value) compared with the variance in the measured data (value obtained by the sensor) [34]. In other words, it provides a measure of the goodness of fit of the calculated model between the measured (sensor) and expected (reference value) values. It is an index whose value ranges from −∞ to 1, where NSE = 1 indicates a perfect model fit. Values of NSE greater than 0.50 [35] or 0.65 [36] are often used as a threshold showing a satisfactory result between observed and simulated values.

In the present research, the less restrictive criterion defined by Moriasi et al. [35] was used, in which the following goodness-of-fit evaluation criteria were established: (i) very good: 0.75 < SES ≤ 1.00, (ii) good: 0.65 < SES ≤ 0.75, (iii) satisfactory: 0.50 < SES ≤ 0.65, and (iv) unsatisfactory: SES ≤ 0.50.

Therefore, statistical hypothesis testing for model adequacy was applied using the FITEVAL v1.0 software [36] based on the NSE of the potential (kPa) measured by each sensor. The hypothesis test allows identifying the significance (*p*-value) of the model performance that exceeds a minimum desirable efficiency threshold (NSE ≥ 0.50) [35].

On the other hand, the RMSE allows quantifying the prediction error of the sensors in kPa, while the PBIAS measures the average tendency of the simulated values (reference value) to be higher or lower than the observed values (potential measured by each sensor), expressed as a percentage. The optimal PBIAS value is 0.0 and low values indicate an accurate simulation of the model. Positive values reflect a model underestimation bias and negative values reflect a model overestimation bias [37].

In general, the soil water potential values recommended for different crops are based both on the experience of professionals in the sector, consultants, or suppliers and on the scientific literature [38]. Most of the research carried out for the determination of the optimum water potential for the different crops [39,40,41,42] shows a wide range of soil potential threshold values. The latter suggests that site-specific factors influence the development of different crops [43]. This is why, for the present investigation, average values found in the literature have been taken into account, establishing two suction ranges for which the 3 indices (NSE, RMSE, and PBIAS) will be calculated: (i) 0–100 kPa: range of interest for vegetable and cereal cultivation and (ii) 0–200 kPa: range of interest for hydrological research.

### 2.3. Test 2: Variability (Repeatability and Reproducibility) in Readings

A second test was carried out in which the variability in the readings recorded on each sensor model was quantified by determining the repeatability and reproducibility indices [44].

Parameter repeatability refers to the ability of a sensor to provide identical readings when the measurement conditions are kept constant. Reproducibility, on the other hand, refers to the similarity of measurements made under different conditions [45,46,47]. In this research, reproducibility refers to the variability in recordings between homologous pairs of the same type of sensor.

The test was carried out in sand—a more homogeneous medium than soil—and 18 experimental sensors (9 models × 2 homologous units) were evaluated simultaneously. It is recalled that, due to the fact that only one unit of the Teros 21 Gen 2 sensor was available, this model could not be evaluated in the second part of the experiment. The experiment was repeated 3 times in order to obtain enough iterations for a more robust statistical analysis.

**Figure 4:**
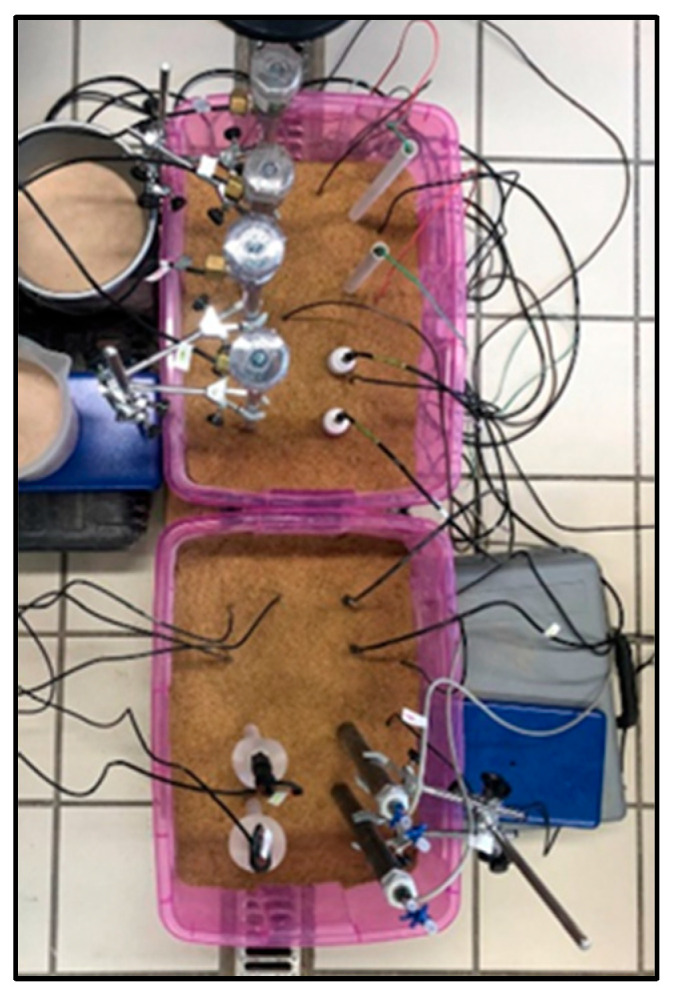
Experimental setup for the repeatability and reproducibility study (Test 2). In two plastic containers (60 × 40 × 30 cm) with sand, the different pairs of homologous sensors were inserted together with humidity probes (see more details in the text).

Experimental design. For the experiments, two 60 × 40 × 30 cm plastic containers (Figure 4) were used, supported at their corners on small blocks, so that the containers were about 5 cm above the floor to facilitate subsequent drainage (see below). For the latter, in addition, the bottom of the container was perforated with small holes and covered with a thin porous cloth. The containers were then completely filled with sand. Each pair of sensors was then carefully inserted into the sand, keeping a minimum separation of about 10 cm between each sensor and between the sensors and the container edge.

The experiment was started with the sand at saturation (0 kPa) and forced drying—with continuous aeration via a small fan—at a drying rate of ~0.3 L·m^−3^·h^−1^. During the test, moisture was measured using 4 volumetric content probes (two in each container). Measurements were taken from each pair of sensors every 24 h for 45 days.

In this second test, it was also necessary to define the SWRC of the sand in order to subsequently, through the moisture readings taken by the 4 probes, be able to infer the corresponding suction value. Unlike Test 1, on this occasion, the retention curve was obtained using the Hyprop instrument (Munich, Germany) [48,49].

The entire experiment, from the filling of the containers onwards, was repeated twice, thus having 3 repetitions in total.

The variability in the suction recordings (repeatability) in the 3 repetitions was determined at 10 pre-set suction/moisture recording points, distributed more or less homogeneously over a suction range from 0 kPa to 180 kPa. In each repetition, each suction–moisture recording point was located, in the respective database, through the average moisture values measured for the 4 moisture sensors, resorting, if necessary, to linear interpolation of moisture values, considering the experimental error inherent to this mathematical operation to be negligible.

Data analysis. The determination of repeatability and reproducibility was carried out by means of an ANOVA [46,50,51]. An ANOVA test was performed for each suction–moisture level, with a significance level of 95%. In this way, the readings recorded by the two homolog units of each sensor for a given suction–moisture level were compared. Also, from the ANOVA test, the variability parameters associated with repeatability (Equation (4)) and reproducibility (Equation (5)) were defined [52]:

(4) $$\sigma_{repeatability}=\sqrt{{MSE}_{ii}}$$

(5) $$\sigma_{reproducibility}=\sqrt{\frac{\left|{MSE}_{ij}-{MSE}_{ii}\right|}{n}}$$

where *σ_repeatability_* and *σ_reproducibility_* are the variability indicators corresponding to repeatability and reproducibility, respectively; *MSE_ii_* is the mean squared error between measurements of the same sensor; *MS_ij_* is the mean squared error between measurements of the two homolog units of the same sensor; and *n* is the number of observations.

The overall variability associated with the sensor will then be:

(6) $$\sigma_{sensor}=\sqrt{{\sigma^{2}}_{repeatability+}{\sigma^{2}}_{reproducibility\;}}$$

The percentages of the repeatability and reproducibility variabilities with respect to the total variability are expressed as:

(7) $$\%\;Repeatability=\frac{{\sigma^{2}}_{repeatability}}{{\sigma^{2}}_{sensor}}\;\cdot 100$$

(8) $$\%\;Reproducibility=\frac{{\sigma^{2}}_{reproducibility}}{{\sigma^{2}}_{sensor}}\;\cdot 100$$

## 3. Results and Discussion

### 3.1. Description of Water Retention Curves

**Figure 5:**
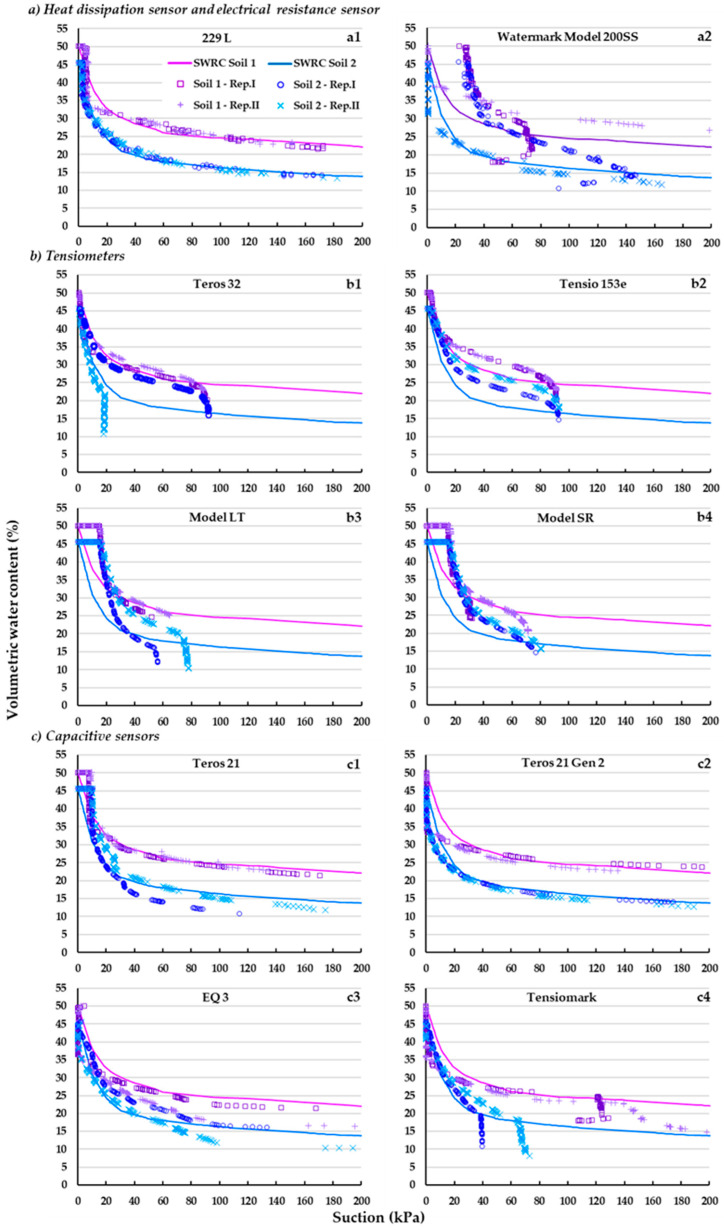
Soil moisture (volumetric water content, %) versus soil matric potential (suction, kPa) of the evaluated sensors (grouped according to operating principle criterion, Figure 1) in each soil (Soil 1 and Soil 1, Table 1). SWRC Soil 1 and SWRC Soil 2: reference water retention curves for Soil 1 and 2, respectively. Rep. I and Rep. II: repetition 1 and repetition 2, respectively.

In the following, the SWRCs obtained with the different sensors and the reference one (control treatment) are presented for both Soil 1 and Soil 2 (Figure 5).

The different curves have a certain parallelism with the reference curve, mainly those corresponding to the heat dissipation sensor and the capacitive ones (Figure 5(a1,c)). In fact, with the exception of the Tensiomark model (Figure 5(c4)), the curves of these two families practically overlap with the reference curve in the suction range from 20 kPa to 150 kPa. The tensiometers—especially in Soil 1—(Figure 5b) show similar behavior to the one just described, but the recording is interrupted—via cavitation—at suction rates of 70–90 kPa [15].

With the exception of the tensiometers Teros 32 and Tensio 153e (Figure 5(b1,b2)), at suctions below 20 kPa (gravitating water), a marked air entry zone is observed in the respective SWRCs, i.e., where the moisture content value remains at saturation within this suction range. This is due to the presence of a more or less high population of occluded or non-functional pores in the porous matrix of the respective sensors, at these low suctions [53]. In contrast, the resistance block and the capacitive ones—with the exception of Teros 21—not only do not show an air entry zone but even register an abrupt drop in moisture content at very low suctions (below 5 kPa), suggesting the existence of a large and functional pore population.

In the case of the heat dissipation sensor, a small plateau is also observed for the same reasons explained above [17,31].

### 3.2. Observed Suction Values vs. Reference Value

**Figure 6:**
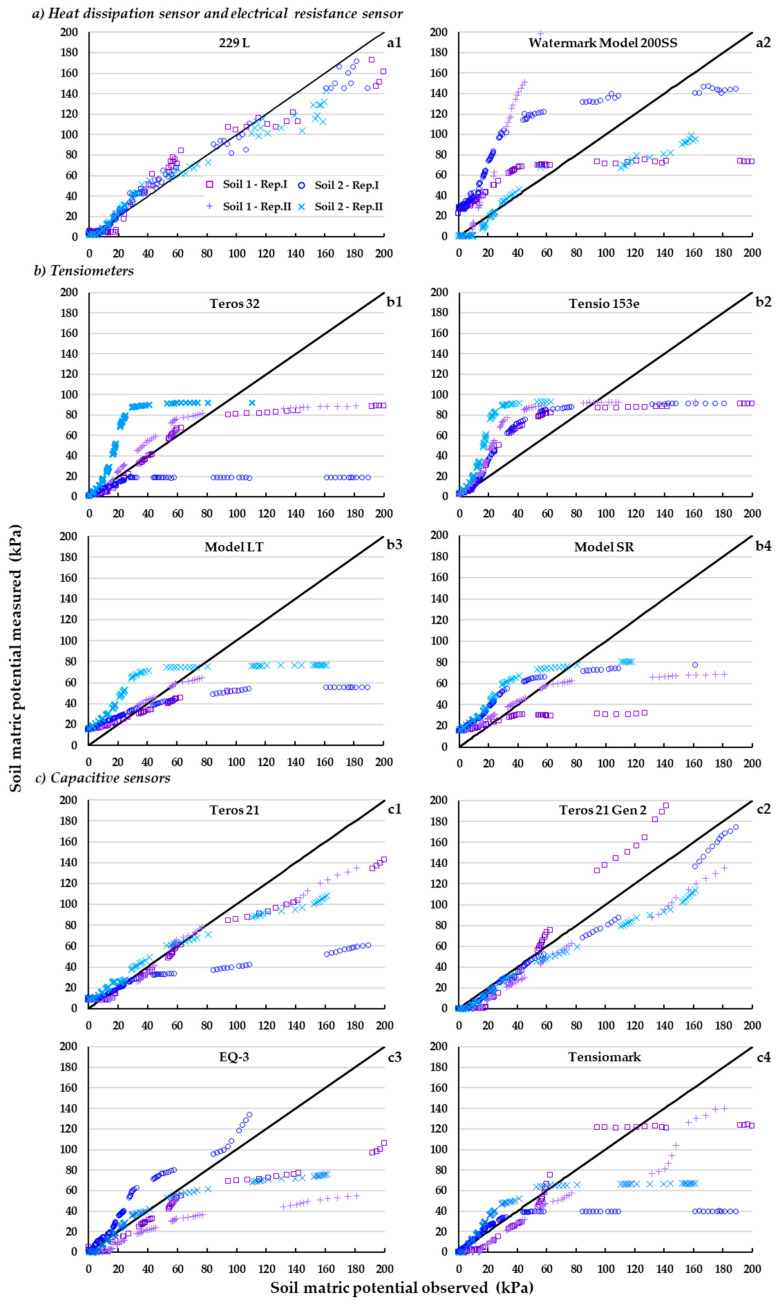
Soil matric potential measured for each type of sensor (Figure 1) versus soil matric potential observed (reference values) in Soil 1 and Soil 2. Rep. I and Rep. II: repetition 1 and repetition 2, respectively.

A clearer way to analyze the performance (reading accuracy) of each sensor in the two study soils is to compare—for different moisture contents—the suction value measured by the device (observed suction) with respect to the corresponding reference value (Figure 6).

In general, the response of the different sensor families was different depending on the suction range (Figure 6).

At suction close to saturation (<20 kPa), only the tensiometer family showed sensitivity, especially the Teros 32 and Tensio 153e models (Figure 6(b1,b2)).

On the other hand, the tensiometers show little or no sensitivity at suctions above approximately 100 kPa (Figure 6b) when cavitation of the tensiometers occurs (see above); while the other tensiometers (capacitive, heat dissipation, and resistance block) are insensitive at suctions below 20 kPa (Figure 6a,c). For example, in the case of the Teros 21 and Teros 21 Gen 2 capacitive sensor, this lack of sensitivity at low suction is due to the fact that the ceramic part of the sensor takes time to start draining the water inside, and the sensor response is not immediate [22]. Similarly, in the work of Malazian et al. [54], which evaluated the overall performance of the MPS-1 sensor, one of the models prior to the Teros 21, data below 30 kPa were discarded because all the sensor units tested started to respond only after exceeding this suction.

Although the heat dissipation sensor and especially the capacitive sensors—unlike the tensiometers—are sensitive to suction above 100 kPa; they underestimate the suction by more than 15% (Figure 6(a1,c)).

On the other hand, between 20 and 100 kPa the capacitive sensors underestimated the suction (Figure 5b), except for the Teros 21 and Teros 21 Gen 2 models, which had a good suction estimation. In contrast, the tensiometers, the heat dissipation sensor, and especially the resistance block overestimated the suction records.

It should be noted that due to a malfunction of the Teros 32 tensiometer (unit 1) during the experimentation on Soil 2, the records from this unit were discarded.

### 3.3. Statistical Analysis

The results obtained in the calculation of the NSE, RMSE, and PBIAS indices for the different suction ranges selected (0–100 kPa and 0–200 kPa) in both soils are shown below (Table 2 and Table 3).

Tensiometers were the only family sensitive to suctions below 20 kPa, making them of particular interest for application in, for example, drip-irrigated crops and small vegetable cultivation [39,42,55].

It is observed that most of the sensors evaluated were not equally accurate in their recordings in both soils. Only the heat dissipation sensor (229-L) and the Teros 21 Gen 2 capacitive sensor performed *very well* to *well* (mean NSE values > 0.9) in both soils, especially between 0–100 kPa; at suction rates of 0–200 kPa, the respective RMSE values of these sensors are almost double those recorded by the same sensors in the lower suction range (0–100 kPa) (Table 2 and Table 3, and Figure 6). However, it should be noted that the bias (PBIAS) in the measurements was much lower—less than half—in the heat dissipation sensor (229-L) than in the capacitive Teros 21 Gen 2. The capacitive Tensiomark also showed little sensitivity to soil type, at least at suction rates below 100 kPa, but with lower accuracy than the two previous sensors (229-L and Teros 21 Gen 2), although equally remarkable (mean NSE values of 0.7–0.8). However, the accuracy of the Tensiomark sensor would be higher for suction not exceeding around 80 kPa, as the sensor starts to cavitate at this threshold (Figure 6). In addition, the 229-L and Teros 21 Gen 2 sensors would have even higher accuracy if we omitted the suction values where they showed no response at all in their readings: 0–20 kPa (Figure 6).

The high sensitivity to soil type shown by the remaining sensors—especially the tensiometer family—is remarkable; for example, the LT tensiometer model showed a mean NSE value of 0.75 or 0.4 and a bias (PBIAS) of +16 or +50, in Soil 1 and Soil 2, respectively (Table 2 and Table 3).

It should be recalled that in this experiment the 229-L heat dissipation sensor had to be calibrated in the laboratory (see above), which would explain, to some extent as just shown, its high accuracy in the measurements in both soils. It was not overlooked, therefore, that those sensors that showed a very satisfactory behavior only in one of the soils analyzed—e.g., the capacitive EQ3 and Tensiomark, Table 2 and Table 3—would also improve their reliability after an ad hoc calibration. Jackisch et al. [12], studying the performance of different water potential sensors in an experimental plot, observed in general a significant inconsistency in the measurements, thus recommending a better calibration of the devices. Similarly, Irmak and Haman [56] concluded that the accuracy of the Watermark granular matrix sensor in sandy soils could be greatly improved through ad hoc calibration equations.

### 3.4. Repeatability and Reproducibility Indices

**Figure 7:**
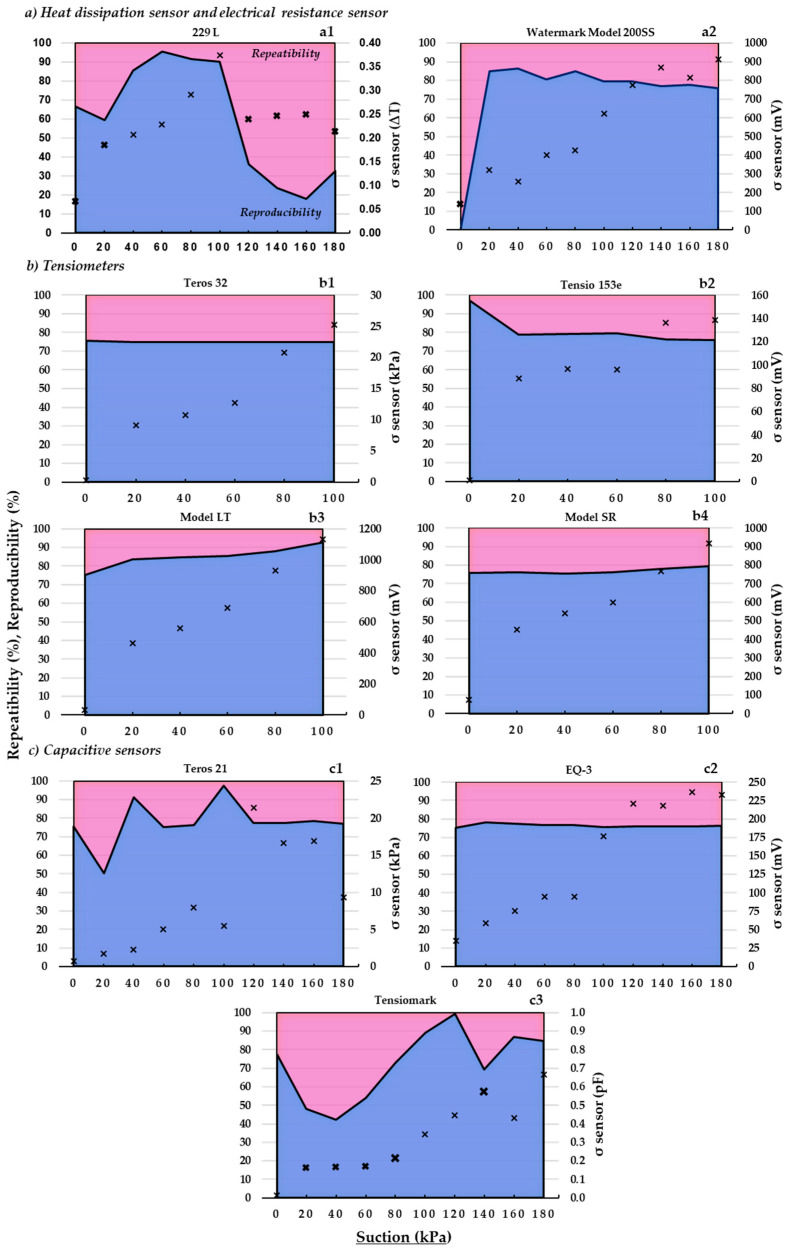
Repeatability (pink) (Equation (7)) and reproducibility (blue) (Equation (8)) (percentages) versus soil matric potential measured (suction, kPa) for each evaluated sensor (Figure 1). σ sensor: the overall variability in the sensor (raw values) (see Equation (6)). The X marker in each graph indicates the overall variability in the sensor where the significant differences (*p* < 0.05) are marked in bold. ∆T: temperature difference; mV: millivolt; pF: Log_10_ hPa.

The repeatability and reproducibility results obtained for each sensor model evaluated—from saturation (0 kPa) to 180 kPa—are shown in Figure 7.

First, we provide some clarifications for the correct interpretation of this figure. The marker (X), besides indicating the value of the overall sensor variability (σ sensor, Equation (6)) at the different suctions, indicates—when it is highlighted in bold—that at that suction value, both units of the same sensor showed significant differences in readings; therefore, they would not have behaved as homologous units. Second, the area below and above the filled line shows, respectively, on the left ordinate, the percentage of repeatability (Equation (7)) and reproducibility (Equation (8)) that, at each suction, make up the total variability in the sensor.

The ANOVA test showed, in general, a high similarity between the values recorded by each pair of homologous sensors over the entire suction range analyzed. Only the 229 L heat dissipation sensor (Figure 7(a1)) and the Tensiomark capacitive sensor (Figure 7(c3)) showed disparity of readings between pairs of homologous sensors at certain suction rates; the disparity of readings between the pair of sensors in the resistance block was not considered relevant, as it only occurred at saturation (0 kPa) (Figure 7(a2)). It is striking that the disparity between pairs of homologous units of the first two sensors mentioned (229-L and Tensiomark) occurred even at suction values where the sensors had (very) accurate recording values, e.g., sensor 229-L at suction values between 120–180 kPa (Table 2 and Table 3). In contrast, it is not surprising that the latter sensor showed the same disparity between homologous units at suctions below 20 kPa as this sensor was, precisely, very insensitive in its readings at these low suctions (see above, Figure 6(a1)).

If we take into account the aforementioned similarity of readings evidenced by most of the homologs of the different sensors, it is not surprising that the total variability in each sensor was due especially to the intrinsic variability in each device (repeatability), rather than, precisely, to the variability among homologous units (reproducibility). More precisely, and, analyzing only the pairs of similar—statistically speaking—sensors, the total variability in the sensors was 75–80% due to repeatability (Figure 7). These results are in agreement with those obtained by González-Teruel et al. [52] who—when evaluating a low-cost soil moisture sensor—determined repeatability values over 75%.

The dispersion of values due to repeatability—i.e., the intrinsic variability in the sensors—could be partly compensated in the field by increasing the number of devices per unit area, without considering the spatial variability in the moisture content/suction of the different soils.

Regarding the overall sensor variability (σ Sensor), most of the devices showed an increase in this dispersion parameter with increasing suction (Figure 7). As the material (sand) containing the sensors dries out (an increase in suction), the sand grains tend to separate since being inert particles their cohesion is only determined by the traction of the capillary forces in unsaturation; thus, the intimate contact of the sensor with the surrounding sand—an indispensable condition for optimal functioning of the sensor—would be gradually limited. It should be noted that this phenomenon would not be (very) different in natural soil—a non-inert material—where the aforementioned contact would also be compromised by the possible appearance of (small) cracks, especially in expansible materials (e.g., Soil 1). These results are in line with what is expected in this type of experiment. For example, in the study by González-Teruel et al. [52], the overall variability in the moisture sensor analyzed acquired minimum values when the soil was at high moisture values.

## 4. Conclusions

The main water potential sensors in use worldwide were evaluated in laboratory conditions in typical agricultural soils of Navarre (Spain). Previously, the large number of existing sensors on the market was classified according to defined operating principles with our own criteria: (i) tensiometers, (ii) capacitive, (iii) heat dissipation, and (iv) resistance block.

The response in terms of accuracy and dispersion of values of the different groups or families of sensors was different according to the suction range considered.

The findings provide information for agricultural and irrigation purposes that could be useful in selecting the most suitable sensor according to different soil types and the specific soil water requirements of different crops. In the agronomically important suction range between 0–100 kPa—which corresponds to available water for plants—the heat dissipation sensor showed the best performance, albeit after an ad hoc calibration. Capacitive sensors have also been shown to be the most accurate. In both families, recordings could be extended to at least 150–200 kPa with approximately the same accuracy, which is interesting for relative water-tolerant deficiency crops—e.g., some grains and seed crops—but also for scientific research. Moreover, these sensors performed similarly in the soils under study—medium to fine textures—and their use would be particularly recommended in sites with different dominant soil types.

In general, the dispersion in readings observed for all sensors evaluated was mainly due (75–80%) to intrinsic sensor variability (repeatability). This should be taken into account when defining the number of devices (replicates) to be installed in the field.

Some sensors, notably the resistance block sensor, would be significantly improved with ad hoc model settings rather than the general ones provided by the manufacturer, although such calibration would be rather difficult to carry out for a conventional user.

In future experiments, it would be useful to quantify the minimum reading time (response time) required by each sensor for a correct reading. For example, a sensor that needs the moisture content to remain constant for a long time to generate a reliable reading would not be suitable for use in surface soil horizons subjected to a high evapotranspiration rate. Moreover, future research is needed to evaluate the sensor performance in field conditions.

A more accurate assessment than the present one could be carried out using data as reference values (suction values for different moisture contents) obtained from Tempe cells instead of from pressure plates.

## Figures and Tables

**Table 1:** Physico-chemical properties of the two soils used in the experiment.

	Soil 1	Soil 2
Sand (Coarse) (%)	7.2	17.3
Sand (Fine) (%)	21.8	29.3
Silt (%)	39.4	39.4
Clay (%)	31.7	14.0
Texture (USDA)	Clay loam	Loam
pH	8.1	7.7
EC (μS-cm)^−1^	176.0	484.0
Organic matter content (%)	2.0	1.9
Carbonates (%)	20.6	40.3
CIC (Cmol-Kg^−1^)	1.7	1.1

**Table 2:** Value of the NSE, RMSE, and PBIAS indices obtained in Soil 1 for each experimental sensor. The RMSE index is in kPa.

**Tensiometers**	**Model SR**	0.59 [0.32–0.79]		16.40 [10.15–24.81]		+6.9%	
	**Model LT**	0.75 [0.64–0.87]		11.93 [8.35–17.74]		+15.85%	
	**Teros 32**	0.84 [0.79–0.90]		14.39 [8.08–20.50]		−7.3%	
	**Tensio 153e**	0.60 [0.06–0.85]		16.21 [10.38–21.19]		+39.8%	
**Capacitive Sensors**	**Tensiomark**	0.81 [0.65–0.89]	0.85 [0.78–0.90]	11.19 [9.33–14.76]	20.30 [11.99–28.49]	−32.0%	−29.1%
	**Teros 21 ** **Gen 2**	0.79 [0.61–0.86]	0.84 [0.77–0.89]	11.60 [9.69–14.06]	17.74 [11.97–23.84]	−26.6%	−19.1%
	**Teros 21**	0.95 [0.92–0.96]	0.90 [0.88–0.94]	5.73 [4.48–7.74]	16.18 [8.13–23.89]	No	−13.6%
	**EQ-3**	0.61 [0.33–0.81]	0.51 [0.23–0.74]	15.84 [10.49–24.46]	36.10 [19.74–53.01]	−45.7%	+50.7%
**Electrical ** **Resistance Sensor**	**Watermark Model 200SS**	−8.47 [−18.09–0.09]	−1.83 [−11.40–0.44]	83.28 [35.62–18.68]	85.58 [40.15–20.64]	+197.5%	+134.2%
**Heat ** **Dissipation ** **Sensor**	**229-L**	0.86 [0.76–0.93]	0.93 [0.88–0.95]	9.59 [7.01–12.63]	13.44 [9.57–17.85]	+11.5%	No
	**Suction ** **Ranges**	**0–100 kPa**	**0–200 kPa**	**0–100 kPa**	**0–200 kPa**	**0–100 kPa**	**0–200 kPa**
		**NSE**		**RMSE**		**PBIAS**	

**Table 3:** Value of the NSE, RMSE, and PBIAS indices obtained in Soil 2 for each experimental sensor. The RMSE index is in kPa.

**Tensiometers**	**Model SR**	0.42 [−0.49–0.72]		17.68 [15.85–20.18]		+69.4%	
	**Model LT**	0.38 [−0.53–0.64]		18.33 [13.66–23.00]		+50.3%	
	**Teros 32**	−1.12 [−10.27–0.74]		31.32 [15.31–44.02]		+107.2%	
	**Tensio 153e**	−0.66 [−5.28–0.61]		27.22 [16.89–37.80]		+98.1%	
**Capacitive Sensors**	**Tensiomark**	0.67 [0.48–0.89]	0.38 [0.14–0.68]	13.36 [6.60–21.68]	40.12 [19.20–61.11]	No	−32.6%
	**Teros 21** **Gen 2**	0.91 [0.84–0.93]	0.92 [0.85–0.97]	7.02 [5.30–9.17]	14.84 [7.39–23.59]	−25.3%	−23.4%
	**Teros 21**	0.68 [0.50–0.91]	0.60 [0.32–0.88]	13.18 [5.78–21.94]	32.19 [14.21–52.13]	No	−23.7%
	**EQ-3**	0.78 [0.29–0.93]	−0.01 [−0.84–0.71]	10.81 [5.83–16.44]	51.12 [20.98–86.98]	+22.4%	+21.5%
**Electrical** **Resistance Sensor**	**Watermark Model 200SS**	0.81 [0.18–0.87]	0.75 [0.68–0.95]	7.42 [4.96–12.03]	28.25 [8.18–44.19]	−17.6%	−33.1%
**Heat** **Dissipation** **Sensor**	**229-L**	0.96 [0.88–0.98]	0.956 [0.93–0.98]	4.77 [3.31–6.33]	10.48 [6.20–15.82]	+6.4%	No
	**Suction ** **Ranges**	**0–100 kPa**	**0–200 kPa**	**0–100 kPa**	**0–200 kPa**	**0–100 kPa**	**0–200 kPa**
		**NSE**		**RMSE**		**PBIAS**	

## Data Availability

Data are contained within the article.
